# Hughes Stovin Syndrome, a Rare Form of Behcet's Disease Presenting as Recurrent Intracardiac Thrombus

**DOI:** 10.7759/cureus.7907

**Published:** 2020-05-01

**Authors:** Anupama B K, Casey Tymko, Rogin Subedi, Jaswinder Virk, Debanik Chaudhuri

**Affiliations:** 1 Internal Medicine, State University of New York (SUNY) Upstate Medical University, Syracuse, USA; 2 Anesthesiology, State University of New York (SUNY) Upstate Medical University, Syracuse, USA; 3 Cardiology, Tulane University School of Medicine, New Orleans, USA; 4 Cardiology, State University of New York (SUNY) Upstate Medical University, Syracuse, USA; 5 Interventional Cardiology, State University of New York (SUNY) Upstate Medical University, Syracuse, USA

**Keywords:** hughes stovin syndrome, pulmonary artery aneurysm, cardiac thrombus

## Abstract

Hughes Stovin syndrome (HSS) is a particularly rare disease characterized by multiple pulmonary artery and/or bronchial artery aneurysms with concomitant peripheral venous thrombosis and is believed to be a cardiovascular variant of Behcet’s disease. Intracardiac thrombus occurring as a thrombotic manifestation of HSS is an unusual presentation and represents a challenge in diagnosis and treatment. Here we report a 25-year-old male presenting with recurrent right-sided intracardiac thrombi, in whom pulmonary artery aneurysm was later detected in the clinical course corroborating the diagnosis of HSS and leading to appropriate initiation of immunosuppressive agents. The patient required multiple cardiac surgeries during the clinical course for cardiovascular complications associated with recurrent cardiac thrombus. Unfortunately, the patient was readmitted a year later for massive hemoptysis secondary to pulmonary arterial aneurysm rupture requiring left lower lobectomy. Our case highlights also the significant morbidity, complications, and treatment challenges associated with this potentially life-threatening syndrome, which is intensified in the presence of cardiac involvement.

## Introduction

Hughes Stovin syndrome (HSS) is a particularly rare disease characterized by multiple pulmonary artery and/or bronchial artery aneurysms with concomitant peripheral venous thrombosis, and is believed to be a cardiovascular variant of Behcet’s disease [1-3]. The variant was first described in 1959 by British physicians John Patterson Hughes and Peter George Ingle Stovin. Intracardiac thrombus is a very rare presentation of HSS and represents a challenge in diagnosis and treatment [4]. Here we describe a 25-year-old male presenting with recurrent right-sided intracardiac thrombi, in whom pulmonary artery aneurysm was later detected in the clinical course confirming the diagnosis of HSS.

## Case presentation

A 25-year-old African American male with a medical history of latent tuberculosis presented with intermittent fever, night sweats, persistent cough, fatigue, and unintentional weight loss over a five-month period. At the time of presentation, the patient was actively undergoing anti-tuberculosis treatment regimen empirically prescribed in the setting of constitutional symptoms despite having had a negative acid-fast bacilli (AFB) and sputum culture. On physical examination, the patient was febrile to a temperature 39.4℃, tachycardic with a heart rate of 125 beats per minute, and tachypneic with a respiratory rate of 30 breaths per minute. His blood pressure was 135/60 mmHg. Further physical examination revealed a systolic murmur over the tricuspid area. Complete blood count was remarkable for a white blood cell count of 12,200/ µL, as well as microcytic anemia with hemoglobin and hematocrit of 8.2 g/dL and 25.9%, respectively. The platelet count was 141,000/µL. Renal and liver function tests were within normal limits.

**Figure 1:**
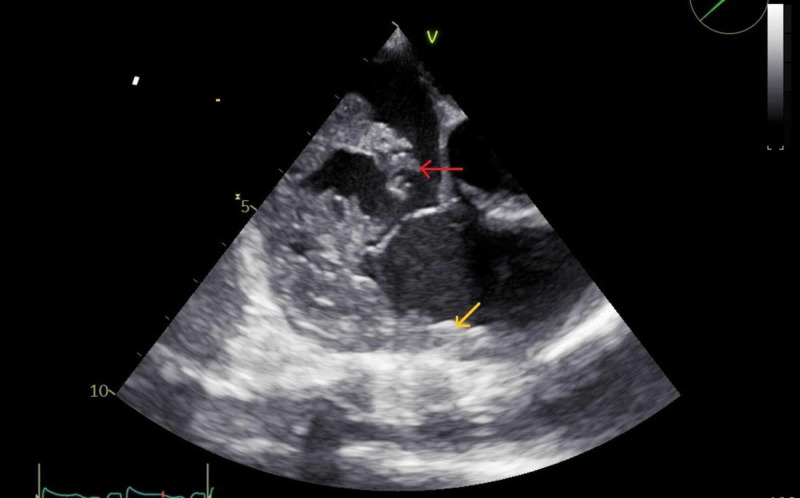
Transesophageal echocardiogram, midesophageal view. The red arrow points to a large ill-defined mobile mass floating in the right atrium. There is extension into the tricuspid valve, subvalvular apparatus, and the right ventricle. The yellow arrow points to thickening of right ventricular free wall.

An initial transthoracic echocardiogram revealed a large ill-defined right-sided intracardiac mass with a subsequent transesophageal echocardiogram (TEE) confirming a 3 cm x 3 cm x 1.5 cm mass extending from the inferior vena cava, into the right atrium, and prolapsing into the right ventricle resulting in severe tricuspid regurgitation (Figure 1).

**Figure 2:**
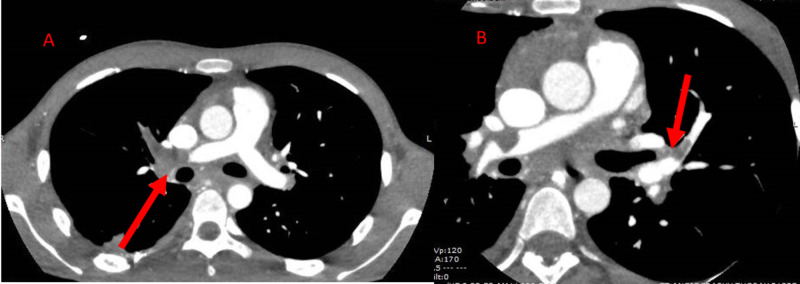
CT angiography of the thorax demonstrating filling defects (red arrows) in the (A) main right pulmonary artery and (B) left upper lobar pulmonary artery suggestive of bilateral pulmonary arterial thrombosis.

At this time, broad-spectrum antibiotic therapy was initiated for possible tricuspid valve endocarditis. The empiric anti-tuberculosis treatment regimen was continued. Thereafter, a cardiac MRI was performed showing diffuse tricuspid valve thickening, multiple enhancing masses on the tricuspid valve leaflets, mass-like thickening at the level of the tricuspid annulus with projection into the right atrium, and diffusely enhancing focal area of right ventricular wall thickening. Initial CT of the thorax revealed bilateral pulmonary arterial thrombosis (Figure 2A-B).

**Figure 3:**
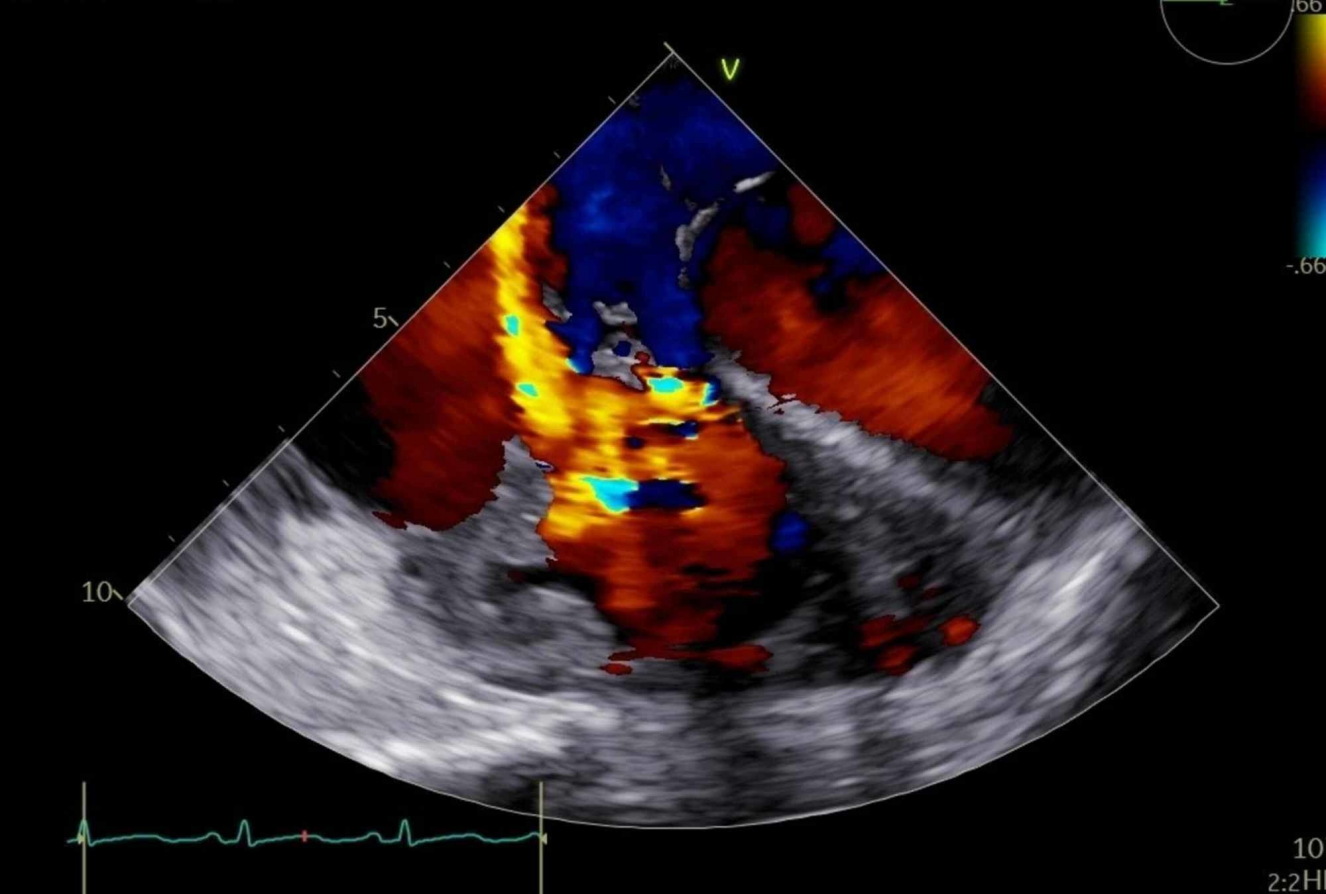
TEE, midesopheageal view, 2D color flow, showing severe tricuspid valve regurgitation. TEE, transesophageal echocardiogram

Ultrasound doppler of the upper extremities showed an acute right distal subclavian, axillary, brachial, and proximal basilic vein thrombosis. At this point, the patient was also started on parenteral anticoagulation. An infectious workup which included blood cultures, AFB stain and cultures, serological tests for HIV, hepatitis B, hepatitis C, Treponema, Epstein Barr virus, Cytomegalovirus, Aspergillus, Histoplasma, Coccidioides, Blastomyces, Bartonella, Tropheryma whipplei, Coxiella, Brucella, and Toxoplasma all returned negative. A bone marrow biopsy did not show any evidence of lymphoma, leukemia, or metastatic tumor. A thrombophilia workup which included analysis for factor V Leiden, and prothrombin gene, Jak2 mutation were negative. An equally extensive rheumatologic workup was also unremarkable. Urine 5-hydroxyindoleacetic acid was negative. Following this extensive workup, the patient underwent right heart catheterization and a TEE-guided endomyocardial biopsy. Histologically, the sample showed detached fibrin with acute and chronic inflammation. There was no histological evidence of granulomas, amyloid deposition, myocarditis, or malignancy. Stains for AFB as well as for fungal and bacterial organisms were negative. A lack of calretinin staining deemed cardiac myxoma less likely. Despite the completion of a full course of empirical infective endocarditis antibiotic therapy and a full anti-tuberculosis drug regimen, the patient continued to decompensate. Repeat TEE performed during this decompensation revealed severe tricuspid regurgitation with perforation of all leaflets (Figure 3).

In the setting of continued severe tricuspid regurgitation and subsequent acute right heart failure, the patient proceeded to emergent cardiac surgery for removal of the right atrial mass and bioprosthetic tricuspid valve replacement. Pathological evaluation of the surgical specimen revealed organizing acute thrombus with inflammatory exudate. Surgical pathology and 16S rDNA analysis were negative for infective etiology. Postoperatively, the patient’s condition improved symptomatically but the diagnosis remained unclear. The patient was medically optimized and discharged on warfarin therapy.

**Figure 4:**
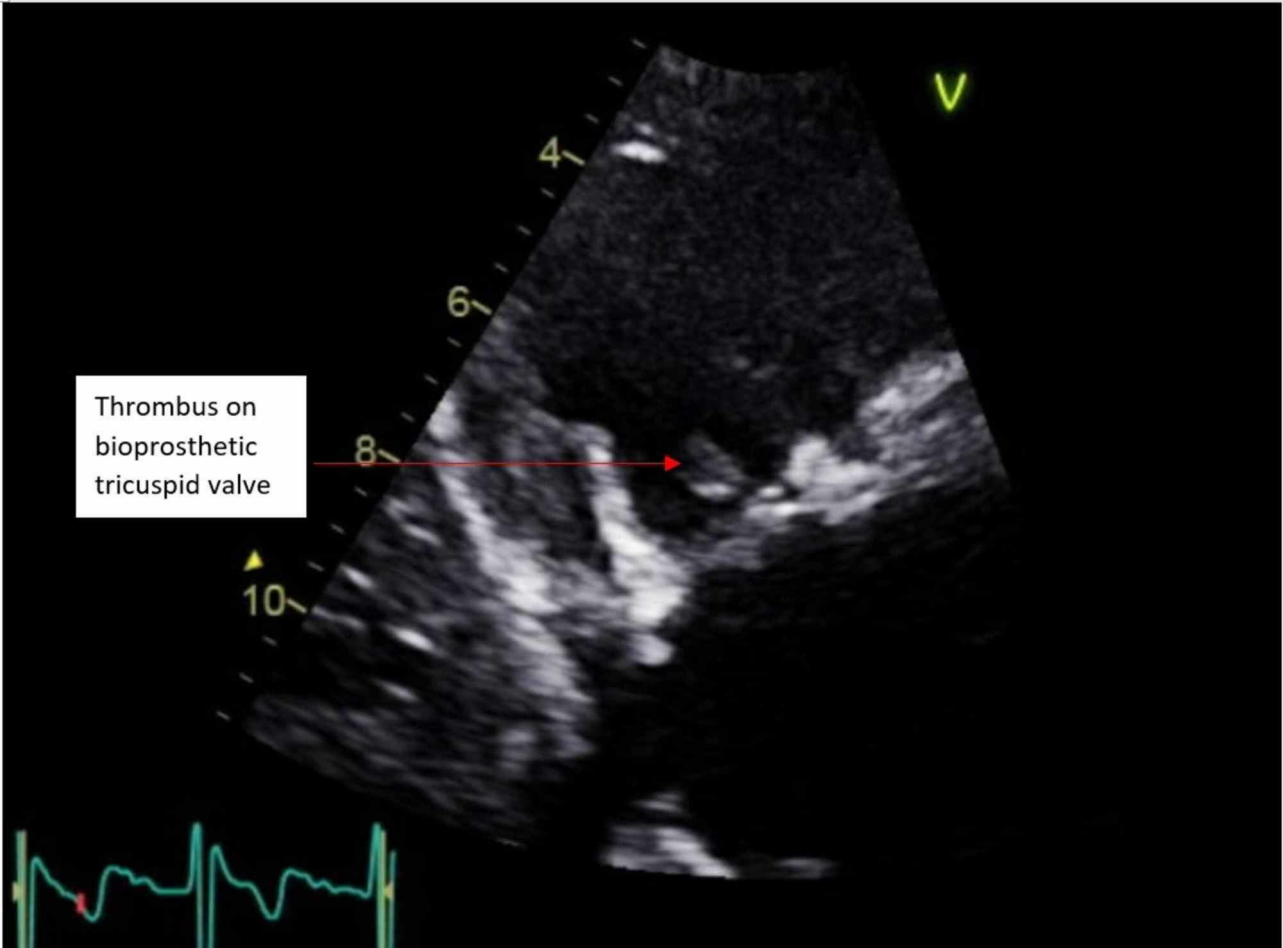
Transthoracic echocardiogram showing mobile echo density measuring 5 mm x 8 mm attached to the tricuspid valve.

One month postoperatively, the patient presented with a recurrence of fever, cough, and shortness of breath. Repeat transthoracic echocardiography at that time showed a recurrent thrombus attached to the bioprosthetic tricuspid valve (Figure 4).

**Figure 5:**
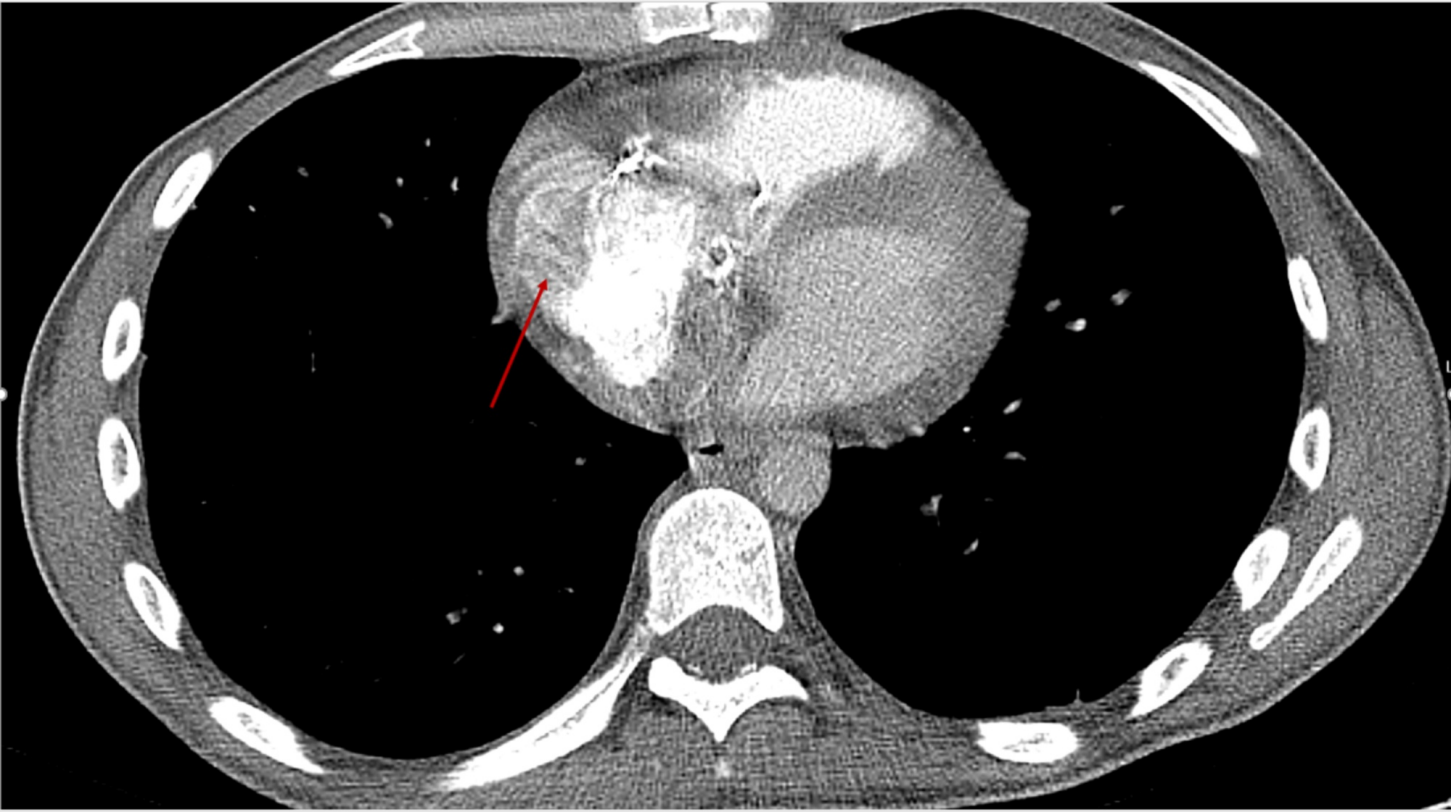
Axial CT thorax with contrast demonstrating recurrent right atrial mass (red arrow).

**Figure 6:**
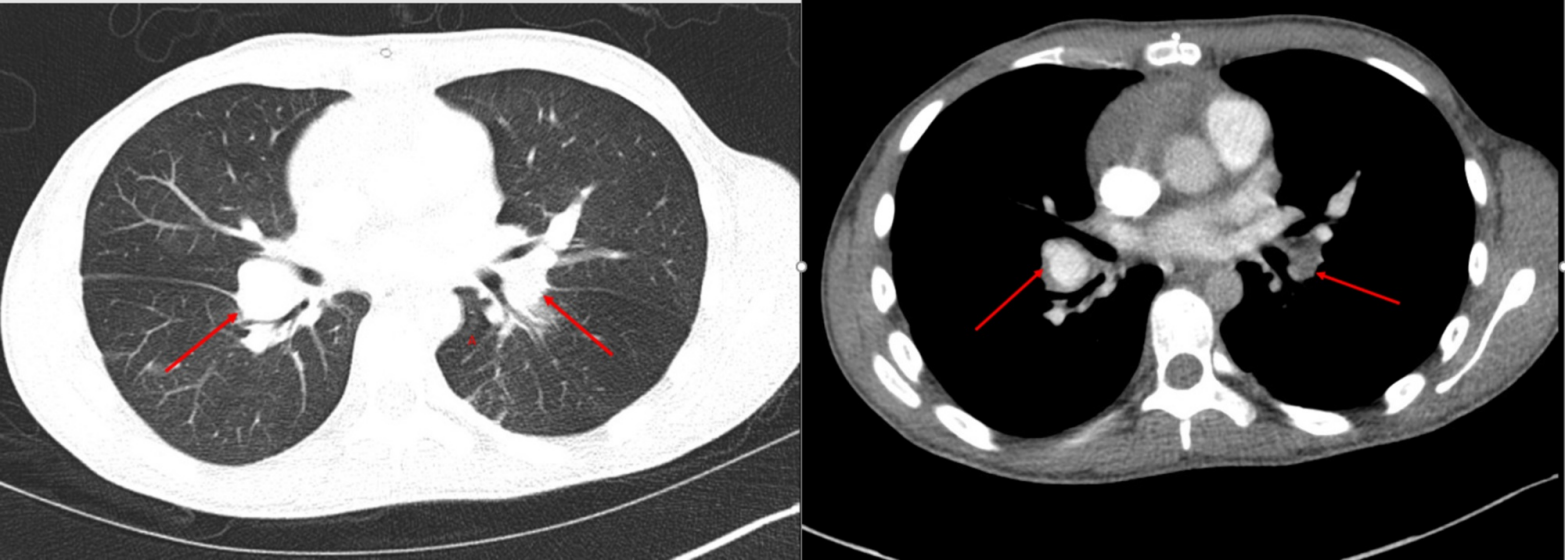
Axial CT thorax with contrast demonstrating bilateral pulmonary artery aneurysms (red arrows).

**Figure 7:**
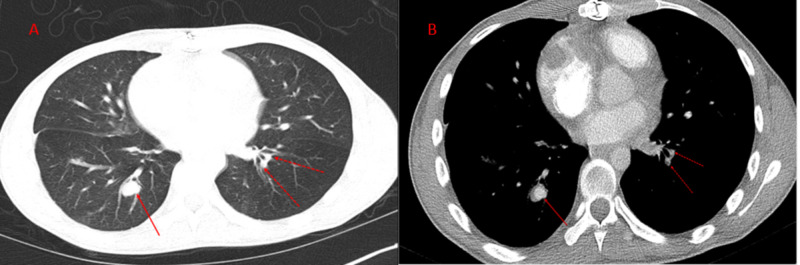
Axial CT thorax with contrast demonstrating bilateral pulmonary aneurysms of the segmental branches of the pulmonary artery (red and dashed arrows).

CT thorax with contrast was repeated showing a recurrent intracardiac right atrial thrombus along with multiple bilateral artery aneurysms, several scattered areas of narrowed and thrombosed pulmonary arteries (Figures 5-7).

**Figure 8:**
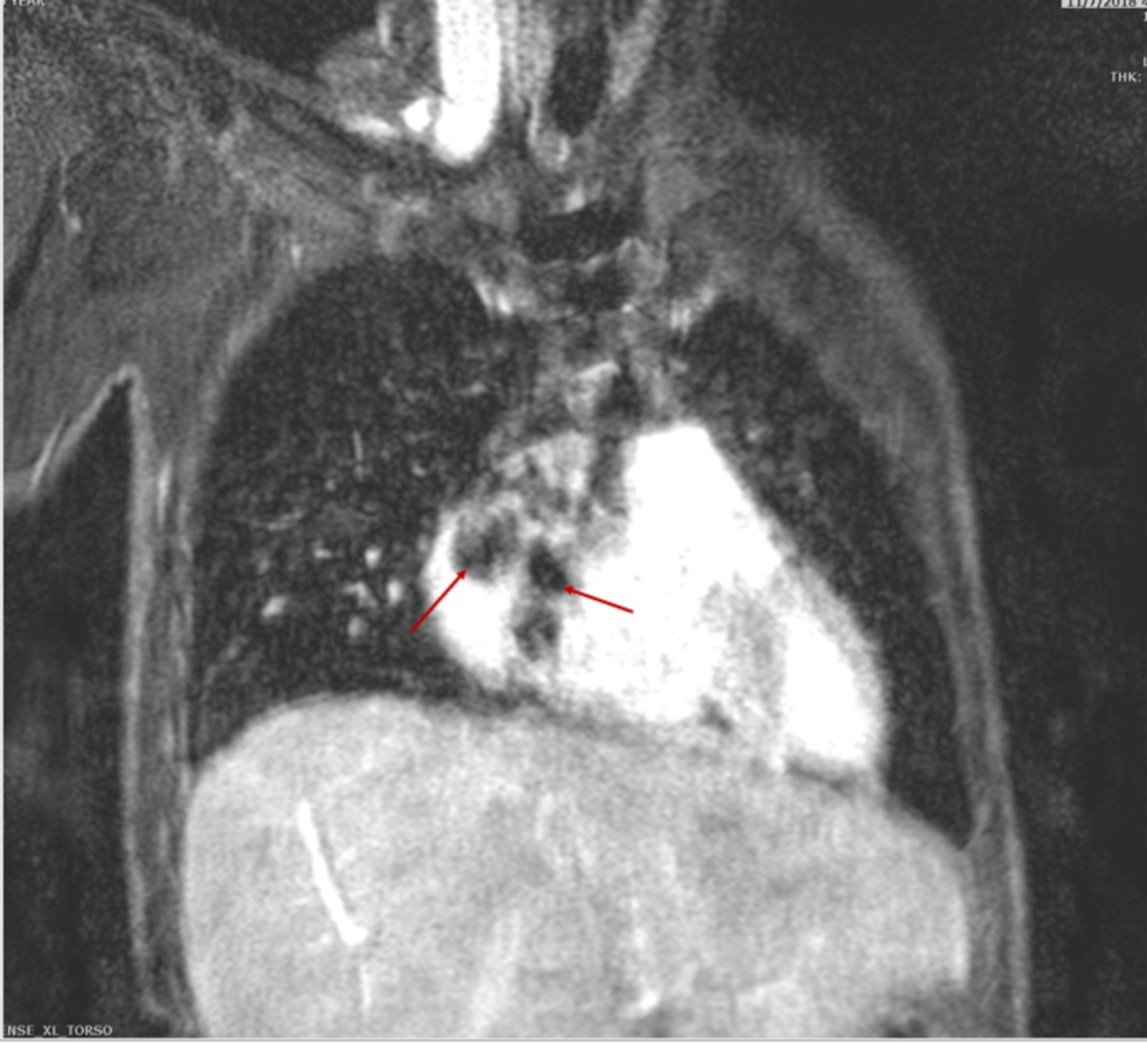
Coronal MRA of the thorax demonstrating filling defects within the right atrial cavity suggestive of right atrial thrombus. MRA, magnetic resonance angiography

**Figure 9:**
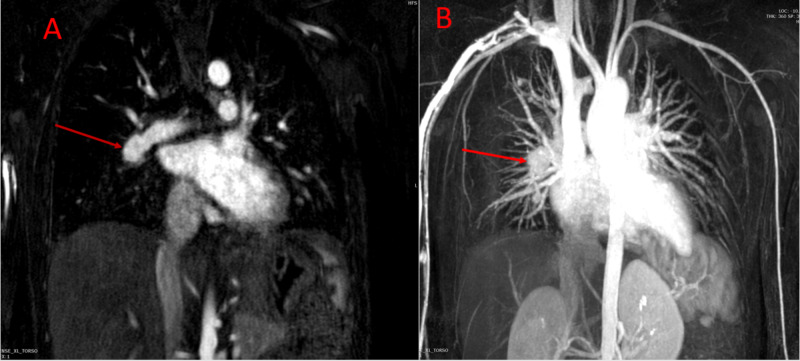
Coronal MRA of the chest demonstrating (A) right main pulmonary aneurysm and (B) 3D MIP image showing right sided pulmonary aneurysm. MRA, magnetic resonance angiography; MIP, maximum intensity projection

**Figure 10:**
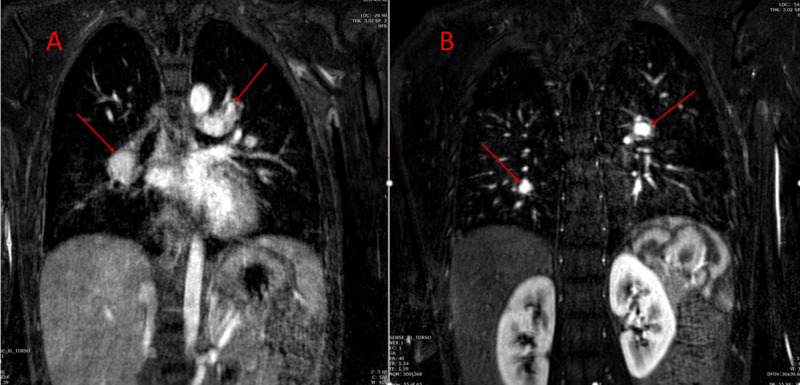
Coronal MRA of the chest demonstrating (A) bilateral pulmonary artery aneurysms (red arrows) and (B) bilateral aneurysms of segmental branches of pulmonary artery (red arrows). MRA, magnetic resonance angiography

**Figure 11:**
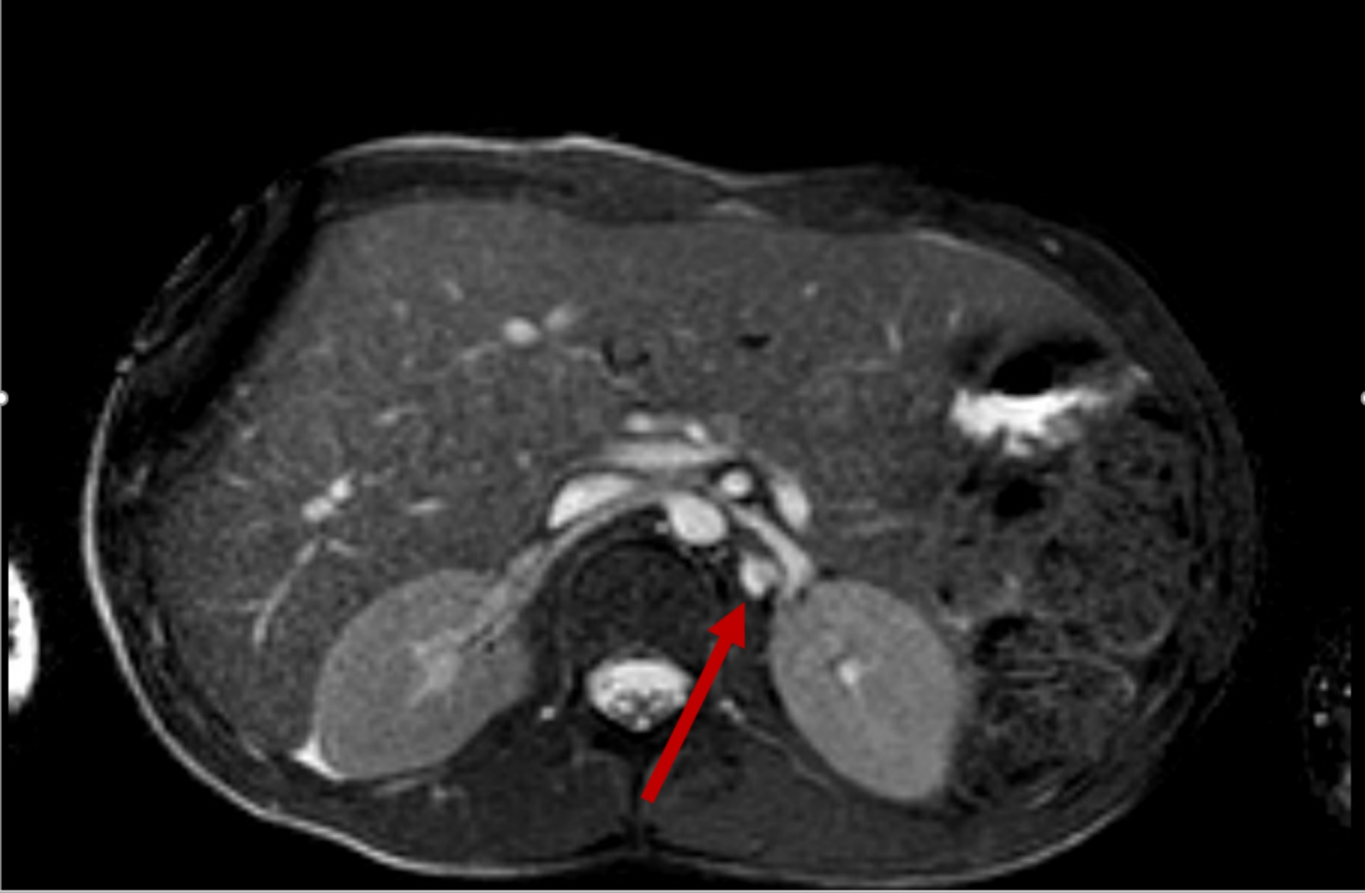
Axial MRA of the abdomen showing diffusely irregular appearance of the left renal artery with an area of focal ectasia in the proximal to mid segment suggestive of an aneurysm (red arrow). MRA, magnetic resonance angiography

**Figure 12:**
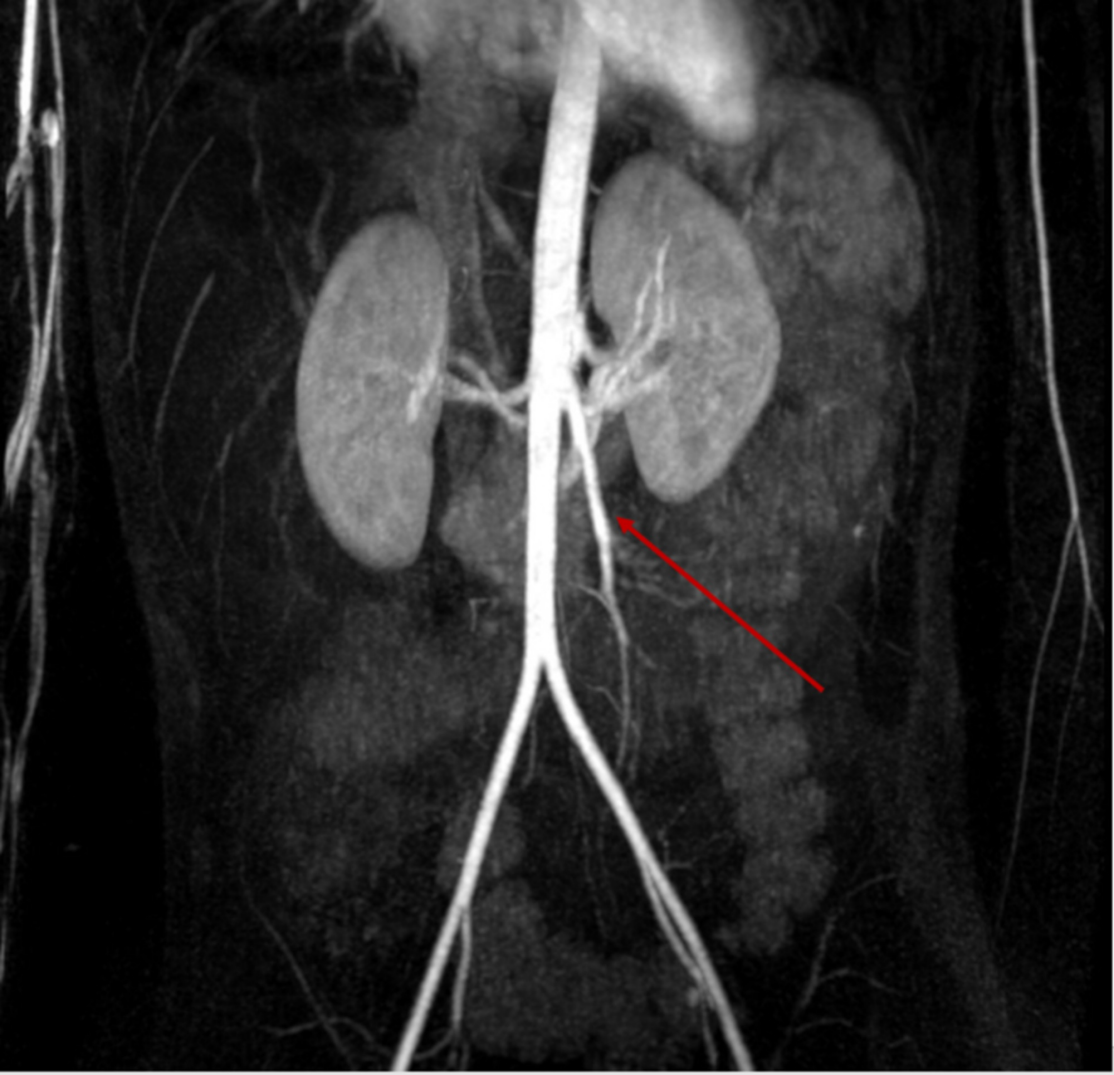
MRA abdomen 3D MIP image demonstrating diffuse irregularity of the superior mesenteric artery. MRA, magnetic resonance angiography; MIP, maximum intensity projection

Magnetic resonance angiography (MRA) of the chest and abdomen further confirmed the presence of right atrial thrombus, focal aneurysms of the right and left pulmonary arteries as well as left renal artery and diffuse irregularities of the superior mesenteric artery (Figures 8-12). 

**Figure 13:**
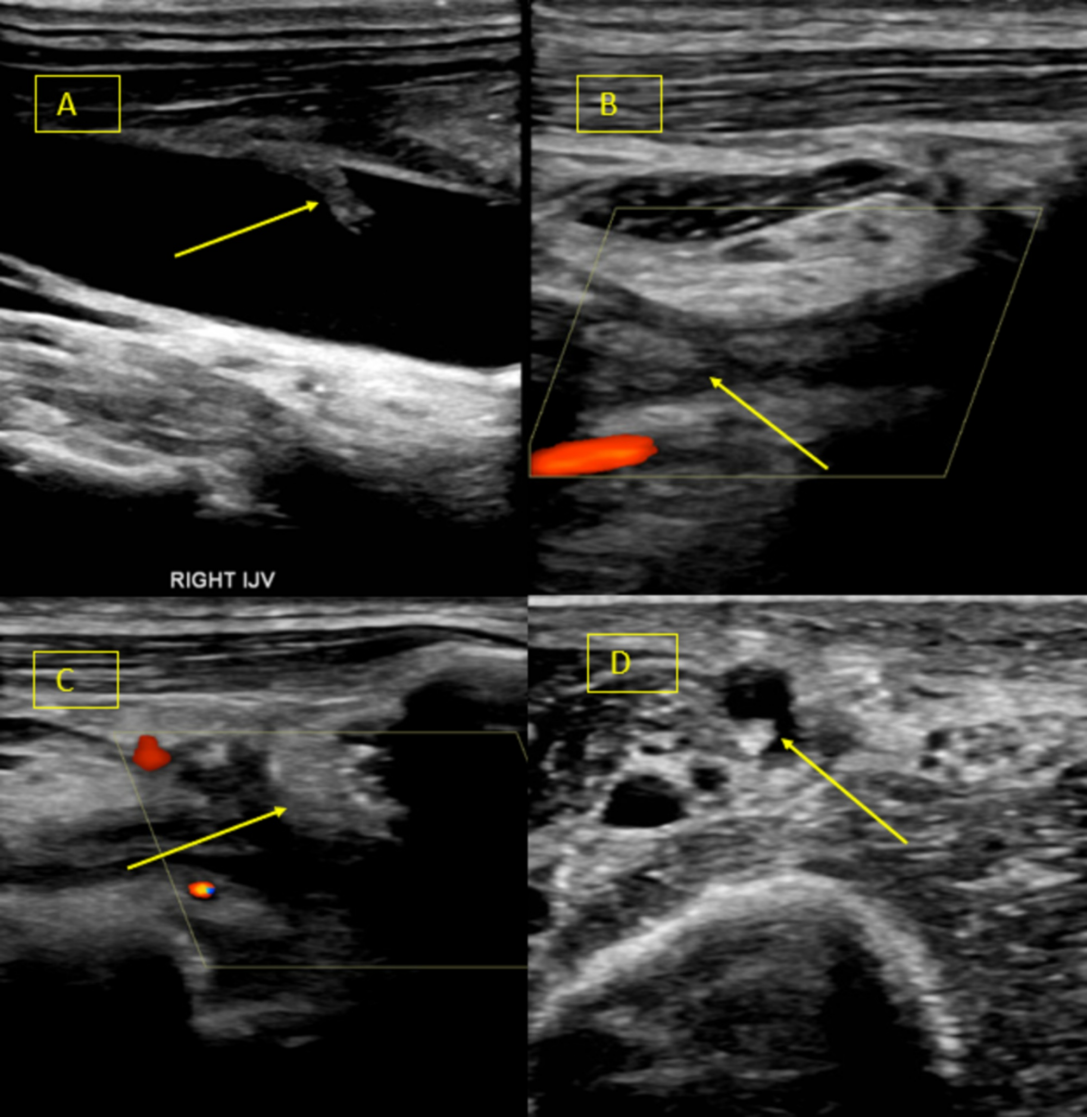
Doppler ultrasound right upper extremity demonstrating (A) chronic, nonocclusive deep venous thrombosis of the right internal jugular vein, (B) chronic, occlusive deep venous thrombosis of the right axillary vein and (C) right subclavian vein, (D) chronic, occlusive, superficial venous thrombosis of the right basilic vein (yellow arrows represents thrombus).

**Figure 14:**
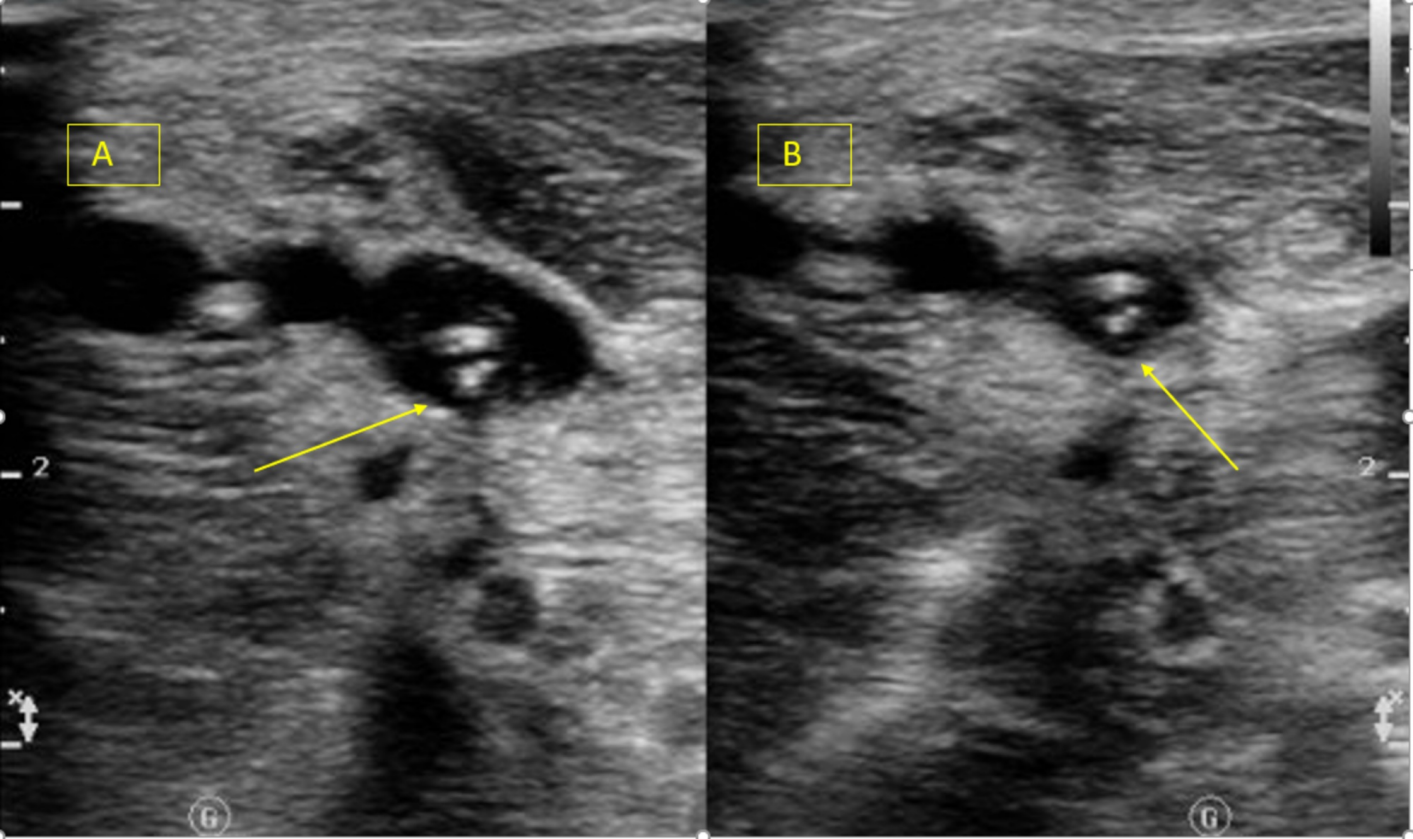
Doppler ultrasound left upper extremity demonstrating acute, occlusive, deep venous thrombosis of the left brachial vein in the mid upper arm (A) thrombosis of the left brachial vein (yellow arrows), (B) noncompressible brachial vein with probe compression.

Ultrasound of the upper extremities showed persistent bilateral deep vein thrombosis (Figures 13-14).

On further questioning, the patient denied any history of ocular, oral, or genital ulcers. Given the constellation of bilateral pulmonary artery aneurysms, biopsy-proven intracardiac thrombi, and peripheral vein thrombosis, a diagnosis of HSS was considered. The right atrial and tricuspid valve masses were considered to be thrombotic manifestations of HSS. A trial of immunosuppression was then initiated with intravenous methylprednisolone 1 g daily for three days and transitioned to oral prednisone 60 mg and oral cyclophosphamide 100 mg daily. The patient was discharged with daily oral cyclophosphamide and prednisone. Cyclophosphamide was discontinued after few months due to thrombocytopenia.

**Figure 15:**
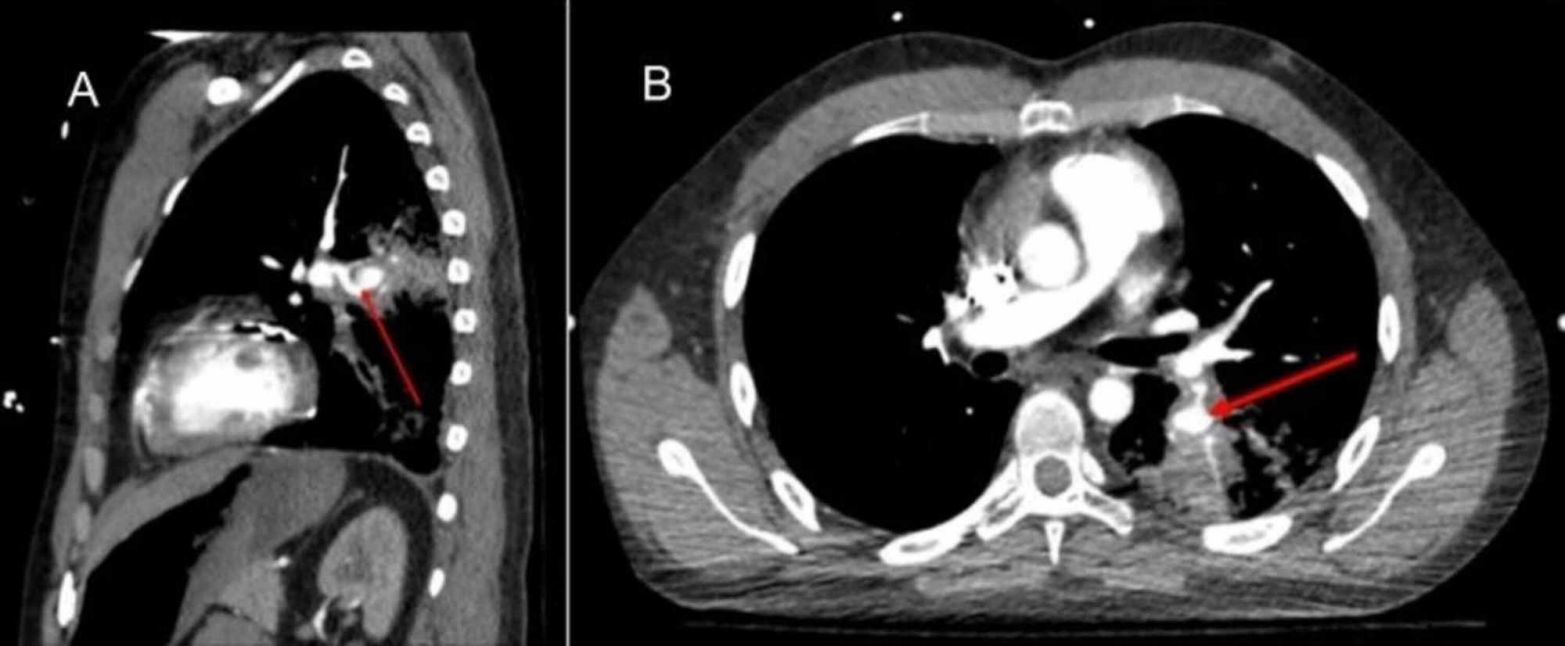
CTA of the thorax, sagittal view (panel A), and transverse view (panel B) showing focal dilatation of the segmental artery in the left lower lobe branch of the pulmonary artery representing a small aneurysm of the left pulmonary artery. Airspace disease is noted in the lung fields. CTA, computed tomography angiography

Five months postdischarge, the patient was readmitted for progressive dyspnea. The patient was found to have complete bioprosthetic tricuspid valve dehiscence with embolization into the right ventricular outflow tract resulting in partial right ventricle outflow obstruction and cardiogenic shock. The patient returned to the operating room for emergent bioprosthetic valve removal and mechanical valve replacement. He was subsequently maintained on oral methylprednisolone, mycophenolate mofetil, and warfarin. A year later, his clinical course was complicated by massive hemoptysis due to left lower lobar pulmonary artery aneurysm (Figure 15 A,B) rupture that required urgent left lower lobectomy. Since then, the patient required a prolonged hospitalization in the critical care unit for acute respiratory failure, hemorrhagic and septic shock but has been slowly making a good clinical recovery.

## Discussion

Hughes Stovin syndrome is typically observed in young males between the second and fourth decades of life [1-3]. Although pulmonary artery aneurysm can be present in both HSS and Behcet's disease, most reported cases of HSS, including the case presented here, were not found to have the mucocutaneous findings of Behcet's disease [1, 5]. The exact etiology and pathogenesis of HSS are unknown, but vasculitis has been proposed as an underlying mechanism [3]. The clinical course of HSS can be divided into three phases which include symptoms of thrombophlebitis, followed by the formation of large vessel pulmonary and bronchial aneurysms, and, lastly, by massive hemoptysis and death secondary to aneurysmal rupture. Aneurysms are commonly observed in the pulmonary and/or bronchial arteries but can be present anywhere in the systemic circulation [3, 6]. Indeed, the patient presented here had aneurysms of the bilateral pulmonary arteries and left renal artery. Recurrent thrombophlebitis and subsequent thrombus formation most commonly affect the peripheral veins, although thromboses of the jugular veins, vena cava, cardiac chambers, and the dural sinus have been described [1, 3]. The patient in this report has intracardiac thrombus, pulmonary artery and deep venous thrombosis. The pulmonary artery thrombosis develops in situ due to the inflammation of the arterial wall and not as thromboembolism developing from peripheral vein thrombosis [7]. Intracardiac thrombus appears to be a result of endomyocardial fibrosis, a sequela of vasculitis involving the endocardium, myocardium, or both, and have been histologically described as an organizing thrombus composed of inflammatory cell infiltrates with a mixture of granulocytes and mononuclear inflammatory cells, as seen in our patient [8]. The presence of pulmonary artery aneurysm carries a very poor prognosis and can be associated with significant mortality due to massive hemoptysis secondary to pulmonary artery aneurysm rupture [3, 5]. However, early diagnosis and timely intervention can significantly improve the prognosis of patients with HSS and can potentially result in disease remission [3].

To date, there are no formal HSS diagnostic criteria or treatment guidelines. The clinical symptoms include fever, cough, chest pain, fatigue, hemoptysis, and dyspnea on exertion and are related to the presence of pulmonary aneurysms and peripheral venous thrombosis. Lab findings are often nonspecific and include leukocytosis, anemia, and elevated erythrocyte sedimentation rate and C-reactive protein. Rheumatological and infectious workup is recommended to rule out competing diagnoses. Diagnosis is usually based on the clinical presentation of venous thrombosis with concomitant pulmonary artery aneurysm in a young patient. A helical CT scan or an MRA is the diagnostic modality of choice for detecting pulmonary artery aneurysms or other visceral arterial aneurysms [3, 6]. Histological studies show diffuse dilatation and partial occlusion of aneurysmal arteries with perivascular lymphomonocytic infiltrate and diffuse proliferative sclerosis [3]. In the case presented, the combination of pulmonary artery aneurysms detected by computed tomography angiography (CTA), recurrent intracardiac thrombus found on 2D echocardiography, TEE, and cardiac MRI, and the confirmation of deep vein thrombosis all led to a clinical diagnosis of HSS.

Despite the lack of formal treatment guidelines, patients are managed similarly to those with Behcet's disease. Management includes immunosuppressive agents such as steroids and cyclophosphamide [1]. IV methylprednisolone 1 g daily is initially given for three days followed by an oral steroid taper. Cyclophosphamide is typically continued for at least one year following complete remission. Anticoagulation alone is an inadequate treatment and the patient may develop recurrent thrombosis despite anticoagulation [2]. Anticoagulant is generally contraindicated because of the increased risk of life-threatening hemorrhage from aneurysm rupture but can be employed with close vigilance in circumstances like presence of cardiac and pulmonary artery thrombosis where the benefit might outweighs the risk [3]. Given the recurrent intracardiac thrombosis and mechanical tricuspid valve placement, our patient was continued on anticoagulation. Surgical options include lobectomy or pneumonectomy which can be performed in the setting of aneurysm confined to one lobe or lung, respectively, or in the setting of massive hemoptysis due to aneurysmal rupture [3, 6]. Transarterial catheter embolization is a strategy reserved for the poor surgical candidate [3]. In the case discussed, a single lobectomy was performed in the setting of aneurysmal rupture confined to a single lobe.

There is no consensus regarding the treatment of cardiac thrombosis. In most cases, cardiac thrombi resolve with anticoagulant, antithrombotic, and immunosuppressive agents. Surgery can become necessary in cases of recurrent cardiac thromboses, thromboses refractory to medical treatment, or massive thrombosis associated with cardiac congestion [9-10]. Although cardiac involvement of HSS is rare, poor prognosis and higher mortality rates in the presence of cardiovascular complications require significant attention [7].

## Conclusions

Hughes Stovin syndrome is a rare type of vasculitis, which is believed to be a cardiovascular variant of Behcet’s disease that presents with venous thrombosis and aneurysms. Although thrombus formation most commonly affects peripheral veins, intracardiac thrombus can occur rarely. HSS should be considered in a patient with intracardiac thrombus in the absence of a clear etiology. These patients should be meticulously investigated for the presence of pulmonary artery aneurysms as the presence of such aneurysms can lead to a clinical diagnosis and hence institution of appropriate treatment. Nevertheless, further studies are required to elucidate the pathogenesis of HSS and to develop formal treatment guidelines.
